# Diverging or converging to a green world? Impact of green growth measures on countries’ economic performance

**DOI:** 10.1007/s10668-023-02991-x

**Published:** 2023-02-08

**Authors:** João J. Ferreira, João M. Lopes, Sofia Gomes, Claudia Dias

**Affiliations:** 1 Department of Management and Economics & NECE-UBI – Research Unit in Business Sciences & QUT Australian Centre for Entrepreneurship Research, University of Beira Interior, Estrada do Sineiro, s/n, 6200-209 Covilhã, Portugal; 2 Department of Management and Economics, Miguel Torga Institute of Higher Education & NECE-UBI – Research Unit in Business Sciences, University of Beira Interior, Estrada do Sineiro, s/n, 6200-209 Covilhã, Portugal; 3 REMIT - Research on Economics, Management and Information Technologies, University Portucalense, R. Dr. António Bernardino de Almeida 541, 4200-072 Porto, Portugal; 4 Research Unit in Business Sciences, NECE-UBI, Universidade da Beira Interior, Estrada do Sineiro, s/n, 6200-209 Covilhã, Portugal

**Keywords:** Green growth index, Green economy, Economic development, Efficient and sustainable resource use, Green economic opportunities, Natural capital protection, Social inclusion

## Abstract

Green growth has emerged in recent years to respond to environmental problems caused by climate change and the scarcity of resources. However, today’s green growth involves environmental, social and financial dimensions. In this context, many countries are currently seeking green growth for their economic development through the efficient use of their resources. This study aims to assess the impact of green growth performance on the economic development of countries. A quantitative approach was applied to a sample of 172 countries worldwide, and the formulated hypotheses were tested through multiple linear regressions estimated by the ordinary least squares method. The economic development of countries was measured by the Human Development Index (HDI) and measures the sustainability performance of countries by the Green Growth Index (GGI). The results of this study demonstrate that (i) the measures of green growth performance have a positive impact on the economic development of high-income, upper-middle-income, and lower-middle-income economies, (ii) in poorer economies, less is the contribution of green growth to economic development, mainly because they neglecting the social dimension despite the numerous existing projects in these economies for greater inclusion and (iii) green economic opportunities (green investment, green trade, green employment and green innovation) do not influence green economic development in all analysed economies. Consequently, suggestions were made for policymakers from different groups of countries to increase and accelerate their sustainable green growth. Literature on economic development and green growth is still scarce, especially at the empirical level, and few studies use the 2020 GGI data. In addition, this study also uses recent rankings of world economies to categorize the economic development of countries.

## Introduction

At the global level, for about three decades, we have witnessed the creation of various instruments and programmes to reduce poverty and enhance sustainable green growth, such as Country Strategy Papers, Poverty Reduction Strategy Papers (PRSPs) and the Millennium Development Goals (MDGs). Thus, these types of programmes have been widely discussed by policymakers, who have increasingly fostered and intensified open dialogue. Thus, green growth-based policies and macroeconomic, social and structural programmes have been developed to promote green growth and reduce poverty, involving all regional actors (Kararach et al., 2018).

Green growth is defined as a path to economic growth, which is based on the use of natural resources in a sustainable manner (Abramovay, 2015; Bagheri et al., 2018). Thus, in recent years, green growth has become increasingly relevant. Politicians have been promoting and warning about the need for economic development and growth based on policies that protect the environment (Bagheri et al., 2018). On the other hand, regions with many natural resources may have serious problems transitioning and transforming to green growth (Cheng et al., 2020b). Natural resources must be managed with criteria in order not to unbalance regional ecosystems (Oliveira et al., 2021).

Thus, combining economic growth and development with environmental protection is not always easy. This new environment for green economic growth may imply restructuring the industry to promote the gradual transformation of green growth. New models are intended to emerge, are increasingly green, and allow reaching the defined goals of carbon neutrality (Zhao et al., 2022). Economic models must stop being climate-change resilient (Dercon, 2014; Houssini & Geng, 2022). To this end, policymakers have been making policy reforms to adjust laws to the needs of today, directing the state budget and its strategies towards sustainable development and accelerating green growth (Akbilgic et al., 2015; D’Souza, 2017). Decisions about sustainable strategies are extremely dynamic and complex because they have to combine energy, environmental and economic measures (Derber, 2015; Schandl et al., 2016). Technological innovation is widely recognized as essential in achieving green growth while reducing poverty. Therefore, technological innovation has to be considered in the constant search for new viable and realistic paths to green growth (Zhao et al., 2022).

Currently, there is no abundant literature about green growth (Kararach et al., 2018; Pan et al., 2019). The years 2011 and 2012 were when the first reports appeared in an attempt to propose some indicators to measure green growth (OECD, 2011; UNEP, 2011). The studies of Kararach et al., (2018), Lyytimäki et al., (2018), Yang et al., (2019) and Šneiderienė et al., (2020) are quantitative studies on green growth, but different indicators are used. So, it can be seen that the application of green growth indices used by researchers is not yet standardised (Zhao et al., 2022), which may lead to different conclusions and recommendations. Thus, scientific knowledge about green growth assessment is still scarce and needs further development (Šneiderienė et al., 2020).

On the other hand, green growth is referred to as an ideal model for developing countries to achieve sustainable economic growth, making it compatible with an increasingly inclusive society and greater environmental protection (Houssini & Geng, 2022). Thus, developing countries face several challenges as natural resources deplete and environmental conditions degrade. From this perspective, policy makers must carry out structural reforms in using natural resources while simultaneously reducing poverty. Therefore, green growth policies must promote the most efficient use of the primary resources they have through technological innovation and the dissemination of knowledge, thus promoting large investments and inclusion (Barbier, 2016).

In this context, in 2019, the “Green Growth Index” was published for the first time by the Global Green Growth Institute. More recently, the green growth index 2020 was published. The Green Growth Index includes 4 dimensions: (1) social inclusion; (2) natural capital protection; (3) green economic opportunities; and (4) efficient and sustainable resource use (Acosta et al., 2019). From this perspective, the sustainable and efficient use of resources has to involve the use of natural capital in a more productive way. It must also produce more economic value using fewer resources, thus not compromising the future wellbeing of society (Acosta et al., 2020a). In this way, it is intended to directly contribute to protecting natural capital (e.g. materials, land, energy, water) and ecosystem services (Flachenecker & Rentschler, 2018; Oliveira et al., 2021).

International organizations, governments, and academia have discussed the growing concerns about green growth (Cheng et al., 2020a; Šneiderienė et al., 2020; Yeh et al., 2019); therefore, the theme is current and pertinent to be studied. In this context, this study aims to assess the impact of green growth measures on the economic development performance of countries. The present study is original since studies encompassing the 2020 Green Growth Index (GGI) data are rare. Moreover, this study also considers the recent classifications of the world economies (high-income countries, upper-middle-income countries, lower-middle-income countries, and low-income countries), which are based on Gross National Income (GNI) per capita. This paper contributes new findings to assess the impact of green growth measures on the economic development performance of high-income, upper-middle-income, lower-middle-income, and low-income countries.

This paper found that green growth performance measures positively impact the economic development of high-income, upper-middle-income, and lower-middle-income countries. The results suggest that the poorer the economies (low-income countries), the less green growth measures contribute to economic development performance. Thus, the poorest economies may be implementing measures that are not reflected in the indicators present in the green growth index. As an alternative to the above, the poorest economies may not value and implement measures that lead to sustainable green development. This article contributes to the development and clarification of the literature on economic development and green growth, a relatively recent topic. We also leave some suggestions for policymakers of different groups of countries to increase and accelerate their sustainable green growth.

## State of the art of green growth and economic development

There is an increasing importance of welfare measurement that goes beyond the analysis of gross national income per capita. In this sense, the Human Development Index (HDI) is a welfare indicator comprising three issues: (1) life expectancy at birth; (2) mean years of schooling and expected years of schooling; and (3) gross national income per capita (Kalimeris et al., 2020). The green growth strategies may positively influence this index, although there are different strategies according to the country’s income level. This depends not only on the particularities of the human, natural and physical capital of the country but also on the environmental culture of each society (Bobylev et al., 2015).

In countries presenting higher-income levels, the focus is the sustainable reduction of per capita environmental footprints, but simultaneously the preservation of human well-being and the mitigation of the in-country inequality while, in countries with lower-income levels, the major question is decreasing poverty and deprivation with inclusive growth and sustainable consumption to meet basic human needs (Luukkanen et al., 2019b). Nevertheless, the majority of residents of big cities around the world, including in low- and middle-income countries, is affected by higher levels of pollutants that originate deadly health effects, decrease labour productivity, boost health expenditures, and diminish crop yields (Akan et al., 2022). In this sense, green growth measures may positively affect the economic development of all countries, regardless of their income level.

### H1

The green growth measures have a positive impact on the economic development of (a) high-income countries, (b) upper-middle-income countries, (c) lower-middle-income countries, (d) low-income countries.

### Efficient and sustainable resource use

One of the dimensions of the GGI is efficient and sustainable resource use. The sustainable use of energy, water, and materials, through, for example, the adoption of renewable energy, efficient technologies or materials reuse, consume fewer resources as well as may produce more outputs not only in higher but also lower-income countries (Shahbaz et al., 2014; Tian et al., 2020), improving human welfare. In the same way, the sustainable use of land is crucial to human, social and economic activities, especially in rural areas where farmland is a critical livelihood resource (Zhang et al., 2020). Even the natural resource abundance of developing countries is not a sufficient condition to improve their economic development since it requires elevated efficiency governance, the rational distribution of resource returns, and the improvement of other growth-oriented measures (Wang et al., 2022).

#### H2

 A efficient and sustainable resource has a positive impact on the economic development of (a) high-income countries, (b) upper-middle-income countries, (c) lower-middle-income countries, and (d) low-income countries.

### Natural capital protection

Another dimension of the GGI is natural capital protection. The decreasing of environmental quality on the planet has been accelerated, not only by greenhouse gas emissions and solid and air pollutants but also by the biogeochemical cycles through land-use change or other natural processes (Liu et al., 2021). Despite developed countries often not depending on natural resource endowment to improve competitiveness, access to natural resources is still the basis for guaranteeing development, influencing their competitive advantage (Li et al., 2021). Hence, to increase human welfare, various governments with different levels of income have recently changed policies to decrease greenhouse gas emissions from only defining carbon abatement targets to promoting various supply and demand-side incentives (Zhang & Wang, 2017), along with biodiversity protection (Bjelle et al., 2021).

#### H3

 A natural capital protection has a positive impact on the economic development of (a) high-income countries, (b) upper-middle-income countries, (c) lower-middle-income countries, and (d) low-income countries.

### Green economic opportunities

Despite the differences between higher- and lower-income countries, green economic opportunities may enhance the countries’ human welfare through green investment, trade, employment and innovation. The positive effects of economic policies promoting green employment can be direct through creating jobs in green areas and indirectly through their contribution to economic growth (Aceleanu et al., 2015). Attending to the increase of greenhouse gas emissions, green trade may be crucial to combat the environmental crisis and increase human welfare (Kang & Lee, 2021), along with green innovation (Choi & Han, 2018) and green investment made by private and public sectors in energy conservation and environmental protection projects (Ren et al., 2020). Albeit creating green economic opportunities, such as the promotion of new green jobs and associated training of workers, requires more financial efforts by developing countries (Bobylev et al., 2015), those opportunities may positively influence the economic development of all economies.

#### H4

 The green economic opportunities have a positive impact on the economic development of (a) high-income countries, (b) upper-middle-income countries, (c) lower-middle-income countries, and (d) low-income countries.

### Social inclusion

Social inclusion is heterogeneous across countries, but their improvement may increase countries’ human welfare through access to basic services and resources, gender balance, social equity and social protection. Developed countries tend to perform better in social inclusion, which impacts their economic development (Li et al., 2021), although social inclusion may also positively influence the economic development of developing countries. To improve human welfare, it is required to increase social equity and social protection and a more equal and just distribution of access to essential services such as water, sanitation, energy, food, transportation and shelter (Thomas, 2019). In the same way, the improvement of the economic power of women has important impacts on society, affecting pensions, health, poverty and fiscal policy (Sánchez et al., 2021).

H5: Social inclusion has a positive impact on the economic development of (a) high-income countries, (b) upper-middle-income countries, (c) lower-middle-income countries, (d) low-income countries.

**Fig. 1:**
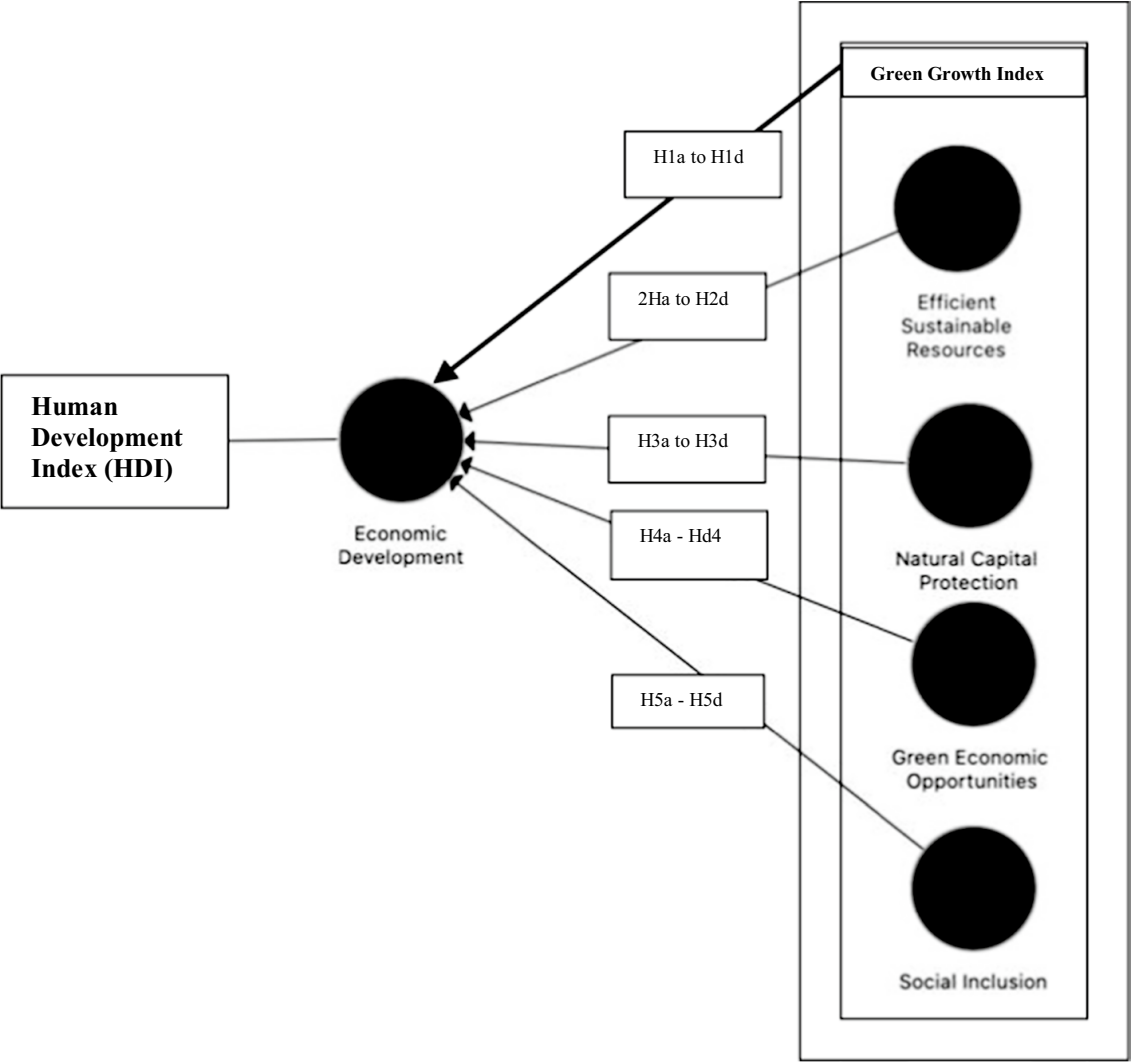
Research framework

The conceptual model and the hypothesis of this study are presented in Fig. 1.

## Methodology and data

### Data

To achieve the objective of this study, data were collected on economic development measured by the 2019 Human Development Index (HDI) collected by the United Nations (United Nations, 2021) and on the measures of green growth performance in the GGGI Technical Report - Green Growth Index 2020 (Acosta et al., 2020b) for the year 2019 (data were consulted in July 2021). This is the second report published on this topic (the previous report was published in 2019, with data from 2018) (Acosta et al., 2019).

According to Acosta et al., (2020b), the GGI is an index that measures the sustainability performance of countries, including the Sustainable Development Goals, Aichi Biodiversity Targets and Paris Climate Agreement. It is measured by four dimensions (green economic opportunities, efficient and sustainable resource use, social inclusion and natural capital protection) and is the first index that measures green growth directly related to sustainable development.

The 2020 green growth index was calculated for 115 countries (21 countries from Africa, 21 from America, 32 from Asia, 38 from Europe and 3 from Oceania) using 2019 data. However, the four dimensions that comprise this index are measured for 186 countries worldwide (48 countries in Africa, 36 in America, 49 in Asia, 42 in Europe and 11 in Oceania). Of these countries, the United Nations made available HDI data in 2019 for 172 countries, and in this way, the database for this study is composed of observations in 2019 for 172 countries (Acosta et al., 2020b).

Following the World Bank classification (Serajuddin & Hamadeh, 2020), the world’s economies will, as of July 1, 2021, be classified in terms of income into four groups based on Gross National Income per capita (GNI per capita) in the current USD: low-income economies with a GNI per capita of $1.045 or below; lower-middle-income economies with a GNI per capita between $1,046 and $4,095; upper-middle-income economies with a GNI per capita between $4,096 and $12,695; and high-income economies with a GNI per capita of $12,696 or above.

According to this classification, the 172 countries that make up the sample were divided into four income groups (the high-income economies group consisting of 57 countries, the upper-middle-income economies group consisting of 45 countries, the lower-middle-income economies group consisting of 50 countries and the low-income economies group consisting of 20 countries (Serajuddin & Hamadeh, 2020). Table 1 shows the statistics (maximum, minimum, mean and standard deviation) of the variables collected to measure economic development and green growth measures by groups of countries.

**Table 1:** Statistics of economic development variables and green growth measures by groups of countries

	Mean	Min	Max	Standard Deviation
*High-income economies*				
Efficient_Sustainable_Resource	4954.400	1224.000	7579.000	1662.412
Natural_Capital_Protection	6672.196	2856.000	8335.000	1425.953
Green_Economic_Opportunities	3766.979	245.000	6384.000	1682.855
Social_Inclusion	7984.537	3882.000	9370.000	1124.795
HDI	0.884	0.778	0.957	0.053
*Upper-middle-income economies*				
Efficient_Sustainable_Resource	4168.049	758.000	7828.000	1310.414
Natural_Capital_Protection	6817.756	3367.000	8484.000	1186.195
Green_Economic_Opportunities	2441.091	359.000	5541.000	1287.510
Social_Inclusion	6504.844	4039.000	8159.000	918.934
HDI	0.761	0.646	0.845	0.047
*Lower-middle-income economies*				
Efficient_Sustainable_Resource	4042.178	984.000	8352.000	1578.998
Natural_Capital_Protection	6609.640	3107.000	8408.000	1148.732
Green_Economic_Opportunities	2058.103	331.000	4834.000	1363.270
Social_Inclusion	4814.700	2036.000	7081.000	1405.214
Green_Growth_Index	3235.931	25.000	5554.000	1681.160
HDI	0.628	0.480	0.783	0.079
Low-income economies				
Efficient_Sustainable_Resource	4220.200	2141.000	7201.000	1491.117
Natural_Capital_Protection	6603.150	4054.000	8455.000	1121.206
Green_Economic_Opportunities	1485.500	398.000	2710.000	956.821
Social_Inclusion	2853.200	1459.000	3948.000	671.531
HDI	0.482	0.394	0.574	0.047

### Methodology

This study uses a quantitative methodology allowing testing relationships between variables and indicators, replicating the methods and techniques applied in other samples and, if the sample size is representative, generalizing the results (Pitkänen et al., 2016; Yin, 2012). In addition, the quantitative methodology allows for more detail in the results, encourages discussion and is more flexible (Qu & Dumay, 2011).

There is still no time series of data on measures of green growth that allows the construction of a panel dataset to estimate multiple linear regressions using the generalized methods of moments. As the data of the variables used were collected only for the year 2019 (smallest sample), linear regressions (single and multiple) were estimated by the ordinary least squares method (OLS) in Eviews 6.0. However, this method has the disadvantage of not being easy to apply to censored data, has optimality properties, and can be quite sensitive to the choice of initial data (Greene, 2020).

As mentioned above, the 2020 green growth index, calculated based on the four measures of green growth (green economic opportunities, efficient and sustainable resource use, social inclusion and natural capital protection), is not published for all countries. In order to obtain the green growth index for all economies in the sample, a cluster or construct was created that aggregates the four green growth measures. The aim is to obtain the green growth index for each country by a group of economies in order to assess the impact of the green growth index on economic development. To this end, with the obtained GGI, a simple linear regression was estimated for each group of economies, which is broadly specified as follows (Eq. 1):

1 $${\text{HDI}}_{i} = c + \beta _{1} {\text{GGI}}_{i} + \mu _{i}$$

where HDI measures economic development (dependent variable) in country i; GGI is the green growth index (independent variable) of country i, being a cluster grouping of the four green growth measures; $\mu$ is the error term; i is the country.

In the second step, the impact of each of the four measures of green growth on the economic development of the four economic groups into which the sample countries were divided was assessed, and four multiple linear regressions were estimated. In generic terms, multiple linear regression is specified as follows (Eq. 2):

2 $${HDI}_{i}=c+ {\beta }_{1}{ESR}_{i}+{\beta }_{2}{GEO}_{i}+{\beta }_{3}{NCP}_{i}+{\beta }_{4}{SI}_{i}+{\mu }_{i}$$

where HDI measures the economic development of country i and is the dependent variable; the independent variables are ESR—efficient and sustainable resource use; GEO—green economic opportunities, NCP— natural capital protection, and SI—social inclusion of country i; $\mu$ is the error term; i is the country.

For the estimation of the multiple linear regressions, it was verified that the following assumptions were met (Greene, 2020): (1) errors (${\mu }_{i}$) are random variables of zero mean; (2) errors (${\mu }_{i}$) are random variables of constant variance (σ2) - hypothesis of homoscedasticity; (3) the random variables are independent; (4) the explanatory variables are uncorrelated - hypothesis of absence of multicollinearity between the explanatory variables; (5) the errors ${\mu }_{i}$ follow a normal distribution. Additionally, a panel data stationarity analysis was performed.

## Results

The results of the stationarity analysis of sample data are shown in Table 2. We conclude that our data are stationary, for mean stationarity is significant at 1%, 5% and 10%.

**Table 2:** Panel unit root tests

Tests variable	Levin-Lin-Chu (2002) - adjusted t*	Breitung (2000) – lambda (statistics)	Im–Pasaran–Shin (1997) – t-tilde-bar (statistics)
Efficient_Sustainable_Resource	−1,9641**	−1,8531**	−1,6536**
Natural_Capital_Protection	−1,8922**	−1,7364**	−1,8034**
Green_Economic_Opportunities	−2,2254***	−1,5654*	−1,7509*
Social_Inclusion	−2,3456**	−1,6345***	−1,8306***
Green Index	−2,6987*	−1,7543**	−1,6649**
HDI	−17,9873 ***	− 1,7385 **	−1,7942 **

Notes: (i) ***, **, * mean stationarity significant at 1%, 5% and 10%; (ii) In all tests, the null hypothesis (H0) is all data panels contain a unit root. (iii) In the case of the Levin–Lin–Chu (2002) test and Breitung (2000) test, we have used a time trend for all variables; In the case of the Im–Pasaran–Shin (1997) test, we have used the time trend for all variables and subtracted cross-sectional means for Efficient Sustainable Resources, Natural Capital Protection, Green Economic Opportunities, Social Inclusion and Green Index.

The first group of simple linear regressions estimated by OLS, as described above, aims to assess the impact of the green growth index on economic development measured by HDI by a group of economies. The green growth index is a cluster or latent variable measured by the green growth indicators—green economic opportunities, efficient and sustainable resource use, social inclusion and natural capital protection. The results of the estimation of simple linear regressions are shown in Table 3.

**Table 3:** Results of simple linear regressions by groups of economies

Economies	Variables	Original Sample (O)	T Statistics (|O/STDEV|)	P Values	R^2^	R^2^ Adjusted
High-income economies	Green Growth Index > HDI	0.616	13.690	0.000*	0.980	0.968
Upper-middle-income economies	Green Growth Index > HDI	0.657	8.085	0.000*	0.832	0.819
Lower-middle-income economies	Green Growth Index > HDI	0.789	3.645	0.000*	0.744	0.736
Low-Income Economies	Green Growth Index > HDI	0.303	0.918	0.359	0.692	0.641

Note: * *p* = 0.000

We can conclude that the green growth index measured by the four green growth measures significantly explains the economic development of three groups of economies (high-, upper-middle-, and lower-middle-income economies). More specifically, a 10% variation in the green growth index causes the HDI to vary by 61.6% in high-income economies and 65.7% in upper-middle-income economies. However, it is not significant to explain the economic development of low-income economies. Thus, we can conclude that hypotheses H1a, H1b, and H1c are confirmed, and H1d is rejected. Unlike other countries, the poorest countries do not have the financial capacity to adopt more advanced and efficient technology (Shahbaz et al., 2014). They have serious problems arising from population growth in the context of very scarce resources, gender imbalance, low social equity and social protection, and low purchasing power (Duro et al., 2020). In this way, the priority of these countries is universal access to the most basic and essential levels of resources, justifying their lesser concern with green growth.

Subsequently, as described above, the impact of each of the green growth measures on countries’ economic development was assessed, and a multiple linear regression was estimated for each group of economies. The results of these regressions are described in Table 4.

**Table 4:** Results of multiple linear regressions for each measure of green growth

Economies	Variables	Original sample (O)	T Statistics (|O/STDEV|)	*P* values	*R*^2^	*R*^2^ Adjusted
High-income economies	ESR > HDI	-0.022	0.159	0.874	0.987	0.955
	NCP > HDI	-0.313	2.827	0.005**		
	GEO > HDI	0.151	1.189	0.235		
	SI > HDI	0.842	5.798	0.000*		
Upper-middle-income economies	ESR > HDI	0.437	2.508	0.012**	0.810	0.861
	NCP > HDI	−0.292	1.488	0.137		
	GEO > HDI	0.188	1.620	0.106		
	SI > HDI	0.631	6.694	0.000*		
Lower-middle-income economies	ESR > HDI	−0.069	0.498	0.619	0.766	0.736
	NCP > HDI	−0.003	0.036	0.972		
	GEO > HDI	0.073	0.940	0.347		
	SI > HDI	0.777	12.067	0.000*		
Low-income economies	ESR > HDI	−0.074	0.190	0.849	0.707	0.731
	NCP > HDI	0.161	0.512	0.609		
	GEO > HDI	0.212	0.926	0.355		
	SI > HDI	0.176	0.460	0.646		

Note: * *p* = 0.000; ** p* <* 0.05

We can conclude that although the green growth measures, when aggregated into clusters (forming the green growth index), are explanatory of economic development, the results change when their impact is assessed separately on the HDI. In high-income economies, natural capital protection and social inclusion are explanatory of economic development, but with different impacts. While natural capital protection has a negative impact on HDI (*ß* = −0.313), social inclusion has a positive impact on HDI (ß = 0.842). Thus, the 5Ha hypothesis is confirmed, and the 3Ha hypothesis is rejected.

The efficient and sustainable resources have a negative impact on HDI (*ß* = −0.022), and the green growth opportunities have a positive impact (*ß* = 0.151), although these variables are not significant in high-income economies. Therefore, we reject hypotheses H2a and H4a. In upper-middle-income economies, efficient and sustainable resource use and social inclusion are explanatory of economic development, both positively impacting HDI (*ß* = 0.437 and *ß* = 0.631, respectively). Thus, hypotheses H2b and H5b are confirmed.

Natural capital protection has a negative impact on HDI (*ß* = −0.292), and green economic opportunities have a positive impact (ß = 0.188) in upper-middle-income economies, but they are not significant in explaining the economic development of this type of economies, rejecting hypotheses H3b and H4b. In lower-middle-income economies, only social inclusion is explanatory of economic development, positively impacting the HDI (*ß* = 0.777). Thus, hypothesis H5c is confirmed.

Also, in lower-middle-income economies, efficient and sustainable resources and natural capital protection have a negative impact on HDI (ß = -0.069 and ß = -0.003, respectively), and green economic opportunities have a positive impact on HDI (*ß* = 0.073), although they are not significant in this type of economies (hypotheses H2c, H3c and H4c are rejected). In low-income economies, none of the green growth measures explain economic development in this group of countries. The efficient and sustainable resource has a negative impact (*ß* = −0.074), and natural capital protection, green economic opportunities and social inclusion have a positive impact (*ß* = 0.161, *ß* = 0.212 and *ß* = 0.176, respectively), although they are not significant (rejects H2d, H3d, H4d and H5d).

Generally, we can conclude that the poorer the economy, the less green growth measures contribute to economic development. This may be because, in these economies, priorities are still focused on other sustainable development goals that the green growth index does not yet comprehensively assess in this type of economy. On the other hand, green economic opportunities are not significant in explaining economic development in all economies. This may result from the fact that this green growth measure, which encompasses green investment, trade, employment and innovation, is more focused on measuring economic growth, neglecting the social aspect. Social inclusion is a measure of green growth that is significant in all economies except low-income economies, where the road to social inclusion is still a difficult battle to win, despite the numerous projects that exist in these economies on this topic.

## Discussion

The results of this study point out that the positive impact of the global green growth index on the HDI is significant in almost all economies except for the low-income economies. In those countries, the separate effect of each dimension related to the green growth index is not also significant. Despite several low-income countries improving their human development significantly over the last years while maintaining a small ecological footprint, it seems the majority increase HDI while also increasing their ecological footprint (Moran et al., 2008). Hence, those countries should use green growth to improve economic and development outcomes, although implementing alternative green pathways will require adequate supporting policies (Luukkanen et al., 2019b).

Resources consumption is necessary for the economic development of all countries, although it may increase emissions of greenhouse gases (Adedoyin et al., 2021). This study shows that the effect of efficient and sustainable resources on HDI is only significant in upper-middle-income economies since the use of resources is different accordingly to the economic activities of each country. Hence, lower-middle-income and low-income economies are based largely on the agriculture sector, while high-income economies are based on the service sector, which is associated with lower use of resources as energy when compared to the industry sector, which mostly supports upper-middle-income countries (Shahbaz et al., 2014). In this way, previous studies already concluded the increase in efficiency of particular resources in middle-income countries is the reduction of cropland demand per food consumption (Duro et al., 2020). The adoption of advanced technology allows more sustainable use of resources, but the higher- and upper-middle income countries have more financial capacity to invest in this technology (Shahbaz et al., 2014). The main sustainability challenge is for low-income countries facing population growth, low agricultural yields and purchasing power (Duro et al., 2020).

In high-income economies, natural capital protection has a significant but negative effect on HDI, which may reflect the higher costs of their policies directed to the protection of biodiversity, greenhouse gas emissions reductions and the improvement of environmental quality (Bjelle et al., 2021), while the economic development impacts may be felt only a long run. According to Li et al., (2021), although the most evolved economies do not essentially depend on natural resources, access is still essential to ensure economic development and maintain competitive advantages. Attending that this specific green growth measure may not be enough to increase HDI in countries where environmental limits have already been largely exceeded, it will be required a combination of measures like substitution of industries, efficiency focus and sustainable consumption (Cibulka & Giljum, 2020). In the other groups of economies, natural capital protection does not significantly impact HDI, contradicting the positive contribution to economic development identified by Zhang & Wang (2017) and Liu et al., (2021). The role of income has a significant impact on the policies adopted, with high-income countries promoting incentives to reduce carbon emissions, while middle-income countries tend to decrease carbon emissions by a policy of transferring economic structure, and low-income countries focus on mandatory measures such as regulations and targets (Zhang & Wang, 2017).

In all groups of economies, the green growth opportunities do not significantly impact HDI. Green employment, innovation, investment, or trade do not seem to present a dimension capable of making a difference in HDI. However, according to Bobylev et al., (2015), green economic opportunities can promote economic development by creating new jobs and greater investment in economies. In this sense, more policies to promote green growth opportunities are essential, although they should focus on economic development goals (Aceleanu et al., 2015). Moreover, green financial policies need to be more stable and continuum since deep fluctuations negatively influence carbon intensity and human welfare (Ren et al., 2020).

Social inclusion has a significant and positive impact on HDI in all economies except for low-income economies. These countries have significantly fewer social inclusion expenditures per capita than others, including access to basic services and resources, gender balance, social equity, and social protection (Ross et al., 2012). The more developed economies have fought over the years for a more inclusive and less discriminating society, influencing their levels of economic development (Li et al., 2021). Furthermore, social inclusion measures are essential to prevent people from falling into poverty (Fiszbein et al., 2014), especially in a period where the impacts of the COVID-19 pandemic affect the groups already in or at risk of poverty (Banks et al., 2021). In the poorest economies, despite the existing advances over time, they still have profound problems of social imbalances in terms of equal opportunities, gender equality, equity, and inclusion (Sánchez et al., 2021).

Countries with higher-income levels may provide more social wealth, resources, and products to their citizens and invest more in environmental protection, which promotes social inclusiveness and economic growth (Li et al., 2021). In this sense, the results of this study show that the impact of efficient and sustainable resources on HDI is only significant in the upper-middle-income economies, while social inclusion has a positive effect on HDI in all economies, excluding low-income economies. Nevertheless, our study concludes that the green growth opportunities do not have significant impacts on HDI, independently of their level of income, distinguishing itself from the studies of Aceleanu et al., (2015) and Kang & Lee (2021). Furthermore, natural capital protection has a negative impact on the HDI of high-income economies. In this sense, there is still a long way to improve green growth outcomes worldwide, especially those related to green growth opportunities (green employment, green innovation, green investment, or green trade) and natural capital protection. This involves enlarging the existing global change research agenda and even creating new tools for measuring green growth (Luukkanen et al., 2019a).

## Conclusion

International organizations, governments, and academia have expressed and discussed their growing concerns about green growth (Šneiderienė et al., 2020). For increasingly green sustainable development to exist, there needs to be development, and for that sustainable development to be long term, it must be accompanied by increased social, ecological, and economic benefits (Lu et al., 2018). In this context, this paper aimed to assess the impact of green growth measures on the economic development performance of high-income, upper-middle-income, lower-middle-income, and low-income countries.

In general, we find that green growth performance measures positively impact the economic development of high income, upper-middle income, and lower-middle income countries. The results indicate that the poorer the economies (low-income countries), the less green growth measures contribute to economic development performance. These economies may not be valuing and implementing measures that lead to green sustainable development, or they may be implementing measures that are not reflected in the indicators present in the green growth index (it is still very recent).

### Policy recommendations

More specifically, the results point out that efficient and sustainable resource positively impacts the economic development of upper-middle-income countries. Thus, high-income, lower-middle-income, and low-income countries have to strengthen their policies regarding material use efficiency, sustainable land use, efficient and sustainable water use, and efficient and sustainable energy. These policies have to consider market coordination, mechanisms available in institutions, industry, science and technology, and rural and urban coordination to create an adequate infrastructure for green growth (Lu et al., 2018).

Regarding natural capital protection, in high-income countries, policymakers have to focus on developing policies that enhance cultural and social value, biodiversity and ecosystem protection, greenhouse gas emissions reductions and environmental quality. Specifically, policymakers have to make considerable investments in renewable energy to reduce environmental quality and greenhouse gas (GHG) emissions. High-income countries must eliminate biodiversity losses due to continued intensive agriculture and deforestation. These countries must be able to reduce emissions from their natural resources, such as gas and oil, and they must also reduce the unsustainable use of their scarce natural resources, such as arable land and fresh water (Acosta et al., 2020a; Fang, 2013).

Concerning green economic opportunities, it is suggested that the policymakers of these countries focus on developing policies in order to increase the rates of green investment, green trade, green employment and green innovation. Thus, they must invest in scientific knowledge and provide incentives for developing green skills. Therefore, business culture must change, and job sustainability guidelines must be given (Grant, 2019). The development and creation of green technology parks can be considered (Oliveira et al., 2021).

Regarding social inclusion, the road to good performance in social inclusion is still long. Policymakers must join efforts to improve and guarantee social protection, social equity, gender balance and access to basic services and resources. Policymakers can provide incentives for creating green companies and even provide benefits for hiring workers for such companies. The waste management sector is an emerging and essential sector for decreasing waste and transitioning to a greener and sustainable economy (Bozkurt & Stowell, 2016; Stowell & Brigham, 2018). Thus, intensive practices should be encouraged, such as composting, recovery, recycling, manufacturing of recycled materials, and transforming waste into energy, eliminating open dumps and landfills (Hajar et al., 2020).

This paper is original, as studies covering the 2020 GGI data are rare. Moreover, this study also considers the recent classifications of the world’s economies (high-income, upper-middle-income, lower-middle-income, and low-income countries), based on Gross National Income per capita (GNI per capita). No known papers assess the impact of green growth measures on economic development performance, taking into account the most recent classifications of the world’s economies. This article contributes to the development and clarification of the literature on economic development and green growth, a relatively recent topic. We also leave some suggestions for policymakers of different groups of countries to increase and accelerate their sustainable green growth.

### Limitations and future lines of research

As limitations, we mention that this study, due to the availability of GGI data, only pertains to green growth performance in 2019. Some countries whose green growth index measures were presented for 2019 in the GGGI Technical Report - Green Growth Index 2020 had to be excluded from the sample due to a lack of data in 2019 and prevented building panel data and calculating regressions applying more robust statistical and econometric techniques. On the other hand, OECD data concerning the green economy could have completed our database, but the lack of updated data collected in recent years made it impossible to include them. The absence of longitudinal data prevented the application of more robust econometric methods, such as the generalized method of moments.

As future lines of research, as soon as more data are available, it is possible to verify the evolution of green growth from year to year (longitudinal study), that is, to build a panel of data with several years and observations for several countries, allowing more robust econometric methods to be applied. We can also test the impact of the sub-variables that make up the four dimensions of the Green Growth Index. The HDI measured human development and in future studies, we can use human development control variables such as average life expectancy, education and GNI per capita. On the other hand, it would be interesting to use a green HDI that directly incorporates the environmental dimension of economies into measuring the economic development of countries. It is also pertinent to understand the impact of green economic opportunities on the economic growth of economies and innovation on green growth performance. Other variables not present in the Green Growth Index can also be considered to study green growth. Studies comparing the green growth of Northern Europe, Western Europe, Central-Eastern Europe and Southern Europe can also be carried out. It is pertinent to study the green growth of peripheral and outermost regions of the European Union. Qualitative and mixed studies can be elaborated, specifically studying which policies for green growth were implemented in each region and their real impacts. New macroeconomic methods for sustainable growth (e.g. green technology and investment, finance, tax) should be tested to help economies become greener. They can also be considered, and taken into account, socio-economic variables that should be the subject of reflection to reduce the deterioration of climate change.

## Data Availability

Data availability statement The data that support the findings of this study are available from the corresponding author, upon reasonable request.

## References

[CR1] Abramovay, R. (2015). *Beyond the green economy*. Routledge. 10.4324/9781315675398

[CR2] Aceleanu MI, Serban AC, Burghelea C (2015). “Greening” the Youth Employment—A chance for Sustainable Development. Sustainability.

[CR3] Acosta, L., Maharjan, P., Peyriere, H., Galotto, L., Mamiit, R., Ho, C., Flores, B., & Anastasia, O. (2019). *Green growth index: concepts, methods and applications* (GGGI technical report, Issue. https://greengrowthindex.gggi.org/wp-content/uploads/2019/10/GGGI_Green_Growth_Index_report.pdf

[CR4] Acosta LA, Maharjan P, Peyriere HM, Mamiit RJ (2020). Natural capital protection indicators: measuring performance in achieving the Sustainable Development Goals for green growth transition. Environmental and Sustainability Indicators.

[CR5] Acosta, L. A., Zabrocki, S., Eugenio, J. R., Sabado, R., Gerrard, J., Nazareth, M., & Luchtenbelt, H. G. H. (2020b). *Green Growth Index 2020 – Measuring performance in achieving SDG targets* (GGGI Technical Report, Issue. https://greengrowthindex.gggi.org/wp-content/uploads/2021/01/2020-Green-Growth-Index.pdf

[CR6] Adedoyin FF, Nwulu N, Bekun FV (2021). Environmental degradation, energy consumption and sustainable development: accounting for the role of economic complexities with evidence from World Bank income clusters. Business Strategy and the Environment.

[CR7] Akan T, Gündüz H, Vanlı T, Zeren AB, Işık AH, Mashadihasanli T (2022). Why are some countries cleaner than others? New evidence from macroeconomic governance. Environment Development and Sustainability.

[CR8] Akbilgic O, Doluweera G, Mahmoudkhani M, Bergerson J (2015). A meta-analysis of carbon capture and storage technology assessments: understanding the driving factors of variability in cost estimates. Applied Energy.

[CR9] Bagheri M, Guevara Z, Alikarami M, Kennedy CA, Doluweera G (2018). Green growth planning: a multi-factor energy input-output analysis of the canadian economy. Energy Economics.

[CR10] Banks LM, Davey C, Shakespeare T, Kuper H (2021). Disability-inclusive responses to COVID-19: Lessons learnt from research on social protection in low- and middle-income countries. World Development.

[CR11] Barbier EB (2016). Is green growth relevant for poor economies?. Resource and Energy Economics.

[CR12] Bjelle EL, Kuipers K, Verones F, Wood R (2021). Trends in national biodiversity footprints of land use. Ecological Economics.

[CR13] Bobylev, S. N., Kudryavtseva, O. V., & Yakovleva, Y. Y. (2015). Regional priorities of green economy. *Economy of Region*, (2), 148–160. 10.17059/2015-2-12.

[CR14] Bozkurt Ö, Stowell A (2016). Skills in the green economy: recycling promises in the UK e-waste management sector. New Technology Work and Employment.

[CR15] Cheng, W. Y., Yang, Z. S., Pan, X., Balezentis, T., & Chen, X. L. (2020a). Evolution of carbon shadow prices in China’s industrial sector during 2003–2017: a by-production approach. *Sustainability*, *12*(2), 10.3390/su12020722.

[CR16] Cheng Z, Li L, Liu J (2020). Natural resource abundance, resource industry dependence and economic green growth in China. Resources Policy.

[CR17] Choi JY, Han DB (2018). The links between Environmental Innovation and Environmental Performance: evidence for high- and Middle-Income Countries. Sustainability.

[CR18] Cibulka S, Giljum S (2020). Towards a Comprehensive Framework of the Relationships between Resource Footprints, Quality of Life, and Economic Development. Sustainability.

[CR19] D’Souza R (2017). Green growth: ideology, political economy and the alternatives. Strategic Analysis.

[CR20] Derber, C. (2015). *Greed to green: solving climate change and remaking the economy*. Routledge. 10.4324/9781315634289.

[CR21] Dercon S (2014). Is green growth good for the poor?. The World Bank Research Observer.

[CR22] Duro JA, Lauk C, Kastner T, Erb KH, Haberl H (2020). Global inequalities in food consumption, cropland demand and land-use efficiency: a decomposition analysis. Global Environmental Change.

[CR23] Fang Y (2013). The effects of natural capital protection on pastoralist’s livelihood and management implication in the source region of the Yellow River, China. Journal of Mountain Science.

[CR24] Fiszbein A, Kanbur R, Yemtsov R (2014). Social Protection and Poverty reduction: global patterns and some targets. World Development.

[CR25] Flachenecker, F., & Rentschler, J. (2018). *Investing in resource efficiency: the economics and politics of financing the resource transition*. Springer. 10.1007/978-3-319-78867-8.

[CR26] Grant R (2019). E-waste challenges in Cape Town: opportunity for the green economy?. Urbani Izziv.

[CR27] Greene, W. H. (2020). *Econometric Analysis: Global Edition*. Pearson.

[CR28] Hajar HA, Tweissi A, Abu Hajar YA, Al-Weshah R, Shatanawi KM, Imam R, Murad YZ, Abu Hajer MA (2020). Assessment of the municipal solid waste management sector development in Jordan towards green growth by sustainability window analysis. Journal of Cleaner Production.

[CR29] Houssini K, Geng Y (2022). Measuring Morocco’s green growth performance. Environmental Science and Pollution Research.

[CR30] Kalimeris P, Bithas K, Richardson C, Nijkamp P (2020). Hidden linkages between resources and economy: a “Beyond-GDP” approach using alternative welfare indicators. Ecological Economics.

[CR31] Kang SJ, Lee S (2021). Impacts of environmental policies on global Green Trade. Sustainability.

[CR32] Kararach G, Nhamo G, Mubila M, Nhamo S, Nhemachena C, Babu S (2018). Reflections on the Green Growth Index for developing countries: a focus of selected african countries. Development Policy Review.

[CR33] Li M, Zhang Y, Fan Z, Chen H (2021). Evaluation and research on the level of Inclusive Green Growth in Asia-Pacific Region. Sustainability.

[CR34] Liu X, Li X, Shi H, Yan Y, Wen X (2021). Effect of economic growth on environmental quality: evidence from tropical countries with different income levels. Science of The Total Environment.

[CR35] Lu N, Wei H, Fan W, Xu Z, Wang X, Xing K, Dong X, Viglia S, Ulgiati S (2018). Multiple influences of land transfer in the integration of Beijing-Tianjin-Hebei region in China. Ecological Indicators.

[CR36] Luukkanen J, Kaivo-oja J, Vähäkari N, O’Mahony T, Korkeakoski M, Panula-Ontto J, Vehmas J, Quoc N (2019). Resource efficiency and green economic sustainability transition evaluation of green growth productivity gap and governance challenges in Cambodia. Sustainable Development.

[CR37] Luukkanen J, Kaivo-oja J, Vähäkari N, O’Mahony T, Korkeakoski M, Panula-Ontto J, Phonhalath K, Nanthavong K, Reincke K, Vehmas J, Hogarth N (2019). Green economic development in Lao PDR: a sustainability window analysis of Green Growth Productivity and the efficiency gap. Journal of Cleaner Production.

[CR38] Lyytimäki J, Antikainen R, Hokkanen J, Koskela S, Kurppa S, Känkänen R, Seppälä J (2018). Developing key indicators of Green Growth. Sustainable Development.

[CR39] Moran DD, Wackernagel M, Kitzes JA, Goldfinger SH, Boutaud A (2008). Measuring sustainable development — nation by nation. Ecological Economics.

[CR40] OECD (2011). *Towards green growth: monitoring progress: OECD indicators* (9264111352). https://www.oecd.org/greengrowth/48224574.pdf

[CR41] Oliveira, J., Lopes, J. M., Farinha, L., Silva, S., & Luízio, M. (2021). Orchestrating entrepreneurial ecosystems in circular economy: the new paradigm of sustainable competitiveness. *Management of Environmental Quality: An International Journal*, *ahead-of*.

[CR42] Pan W, Pan W, Hu C, Tu H, Zhao C, Yu D, Xiong J, Zheng G (2019). Assessing the green economy in China: an improved framework. Journal of Cleaner Production.

[CR43] Pitkänen K, Antikainen R, Droste N, Loiseau E, Saikku L, Aissani L, Hansjürgens B, Kuikman PJ, Leskinen P, Thomsen M (2016). What can be learned from practical cases of green economy? –studies from five european countries. Journal of Cleaner Production.

[CR44] Qu SQ, Dumay J (2011). The qualitative research interview. Qualitative Research in Accounting & Management.

[CR45] Ren X, Shao Q, Zhong R (2020). Nexus between green finance, non-fossil energy use, and carbon intensity: empirical evidence from China based on a vector error correction model. Journal of Cleaner Production.

[CR46] Ross AD, Parker H, Benavides-EspinosaDroge MC (2012). Sustainability and supply chain infrastructure development. Management Decision.

[CR47] Sánchez, R., Finot, J., & Villena, M. G. (2021). Gender wage gap and firm market power: evidence from Chile. *Applied Economics*, 1–13. 10.1080/00036846.2021.1985070.

[CR48] Schandl H, Hatfield-Dodds S, Wiedmann T, Geschke A, Cai Y, West J, Newth D, Baynes T, Lenzen M, Owen A (2016). Decoupling global environmental pressure and economic growth: scenarios for energy use, materials use and carbon emissions. Journal of Cleaner Production.

[CR49] Serajuddin, U., & Hamadeh, N. (2020). *New World Bank country classifications by income level: 2020–2021*. World Bank. Retrieved 12 july 2021 from https://blogs.worldbank.org/opendata/new-world-bank-country-classifications-income-level-2020-2021

[CR50] Shahbaz M, Nasreen S, Ling CH, Sbia R (2014). Causality between trade openness and energy consumption: what causes what in high, middle and low income countries. Energy Policy.

[CR51] Šneiderienė A, Viederytė R, Abele L (2020). Green growth assessment discourse on evaluation indices in the European Union. Entrepreneurship and sustainability issues.

[CR52] Stowell, A. F., & Brigham, M. (2018). Extractivism, value and waste. *Etnografia e Ricerca Qualitativa*, (1), 75–95. 10.3240/89695.

[CR53] Thomas, E. (2019). Toward a New Field of Global Engineering. *Sustainability*, *11*(14), 3789. 10.3390/su11143789.

[CR54] Tian X, Sarkis J, Geng Y, Bleischwitz R, Qian Y, Xu L, Wu R (2020). Examining the role of BRICS countries at the global economic and environmental resources nexus. Journal of Environmental Management.

[CR55] UNEP (2011). *Towards a green economy: Pathways to sustainable development and poverty eradication*. https://sustainabledevelopment.un.org/content/documents/126GER_synthesis_en.pdf

[CR56] United Nations (2021). *Human Development Index*. Retrieved 12 july 2021 from http://hdr.undp.org/en/indicators/137506

[CR57] Wang S, Wang X, Lu B (2022). Is resource abundance a curse for green economic growth? Evidence from developing countries. Resources Policy.

[CR58] Yang Y, Guo H, Chen L, Liu X, Gu M, Ke X (2019). Regional analysis of the green development level differences in chinese mineral resource-based cities. Resources Policy.

[CR59] Yeh SC, Chiou HJ, Wu AW, Lee HC, Wu HC (2019). Diverged preferences towards Sustainable Development Goals? A comparison between Academia and the Communication Industry. International Journal of Environmental Research and Public Health.

[CR60] Yin, R. K. (2012). *Applications of case study research* (3 ed.). SAGE Publications.

[CR61] Zhang D, Wang W, Zhou W, Zhang X, Zuo J (2020). The effect on poverty alleviation and income increase of rural land consolidation in different models: a China study. Land Use Policy.

[CR62] Zhang X, Wang Y (2017). How to reduce household carbon emissions: a review of experience and policy design considerations. Energy Policy.

[CR63] Zhao J, Shahbaz M, Dong K (2022). How does energy poverty eradication promote green growth in China? The role of technological innovation. Technological Forecasting and Social Change.

